# Lung adenocarcinoma with bladder metastasis: A case report and literature review

**DOI:** 10.3389/fonc.2023.1202885

**Published:** 2023-07-05

**Authors:** Yuying Liu, Xinyue Dong, Tao Li, Yanke Xing, Na Liu

**Affiliations:** ^1^ Department of Oncology, Qilu Hospital of Shandong University, Dezhou Hospital, Dezhou, Shandong, China; ^2^ Department of Pathology, Qilu Hospital of Shandong University, Dezhou Hospital, Dezhou, Shandong, China

**Keywords:** metastasis, lung adenocarcinoma, bladder metastasis, secondary tumor, hematuria

## Abstract

**Background:**

Lung cancer is the most common cause of cancer-related mortality in the world. Unfortunately, more than 50% of patients have already metastasized at the time of diagnosis, contributing to morbidity and mortality. Common sites of metastasis are adrenal glands, liver, bone, and brain. Bladder metastasis is rare and should prompt a careful differential consideration of primary bladder cancer.

**Case description:**

Here, we report a 72-year-old female who went to the hospital for “gross hematuria for one day”. Cystoscopy showed space-occupying lesions in the bladder. During the general CT examination, space-occupying lesions in the lower lobe of the lung were found. Peripheral lung cancer with multiple lymph node metastases, pulmonary metastasis, and left pleural effusion were considered. Transurethral cystoscopic resection of the bladder tumor and pleural effusion cell block examination were performed to clarify the diagnosis. Combined with morphological and immunohistochemical results, both pathological results supported a diagnosis of lung adenocarcinoma metastasis, and gene detection was carried out. EGFR, ALK, and ROS-1 were negative. According to the genetic testing results, there was no corresponding targeted drug, so we administered chemotherapy, and one-year survival was achieved, which was better than expected based on other studies.

**Conclusion:**

This paper describes a case of lung adenocarcinoma metastatic to the bladder and includes a review of the literature to provide clinicians with diagnostic and treatment experience and help avoid misdiagnosis and mistreatment.

## 1 Introduction

Bladder metastasis of lung adenocarcinoma is extremely rare, and thus, the diagnosis in such a situation requires careful differentiation from primary bladder cancer. Because the symptoms and imaging findings are similar, differential diagnoses of primary bladder tumors from metastatic tumors should be based on accurate pathologic evaluation and, above all, on specific immunohistochemical staining. A case report of a patient admitted to our department with bladder metastasis of lung cancer is as follows.

## 2 Case report

**Figure 1:**
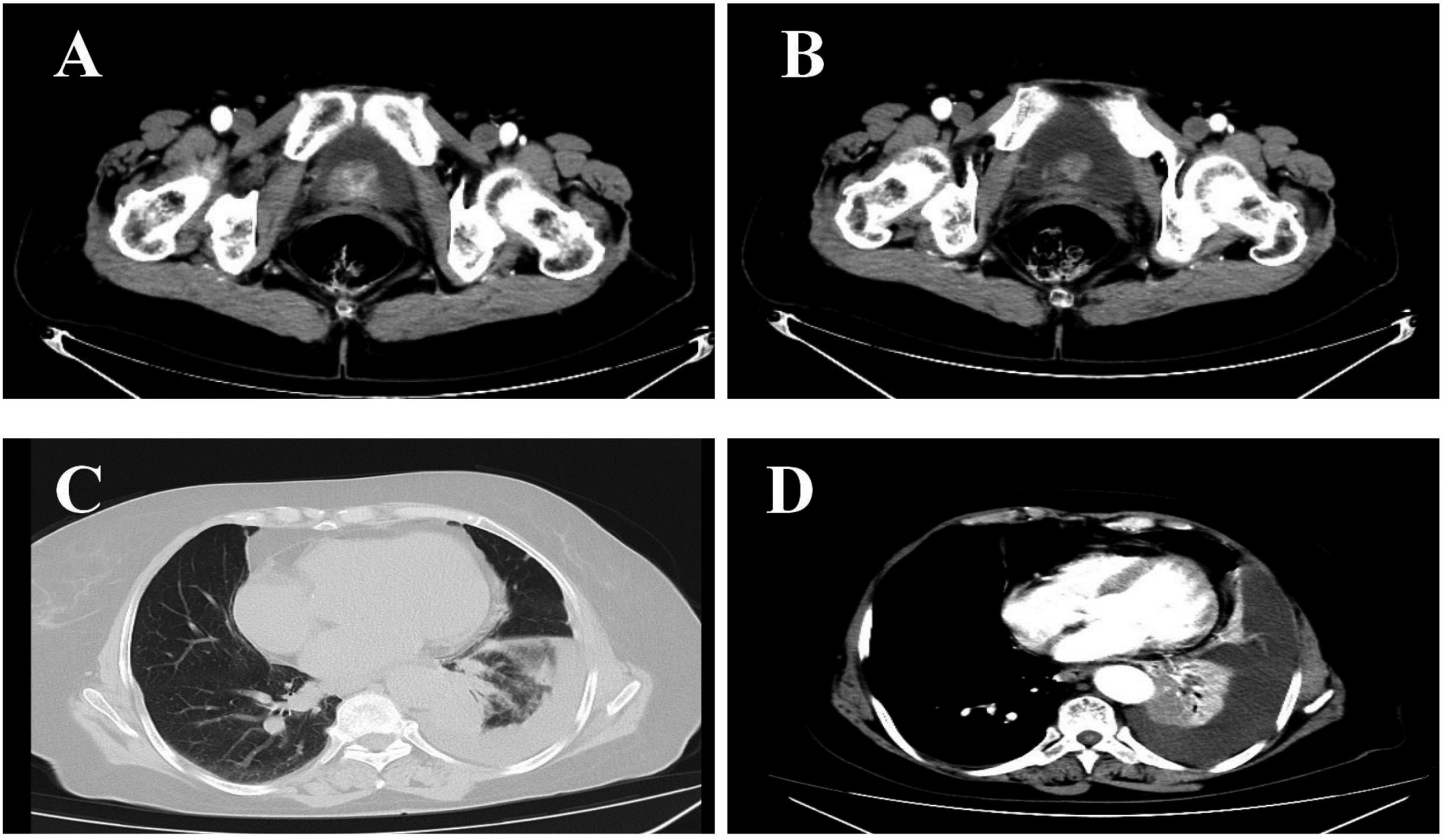
**(A, B)** bladder tumor, **(C, D)** lung cancer.

**Figure 2:**
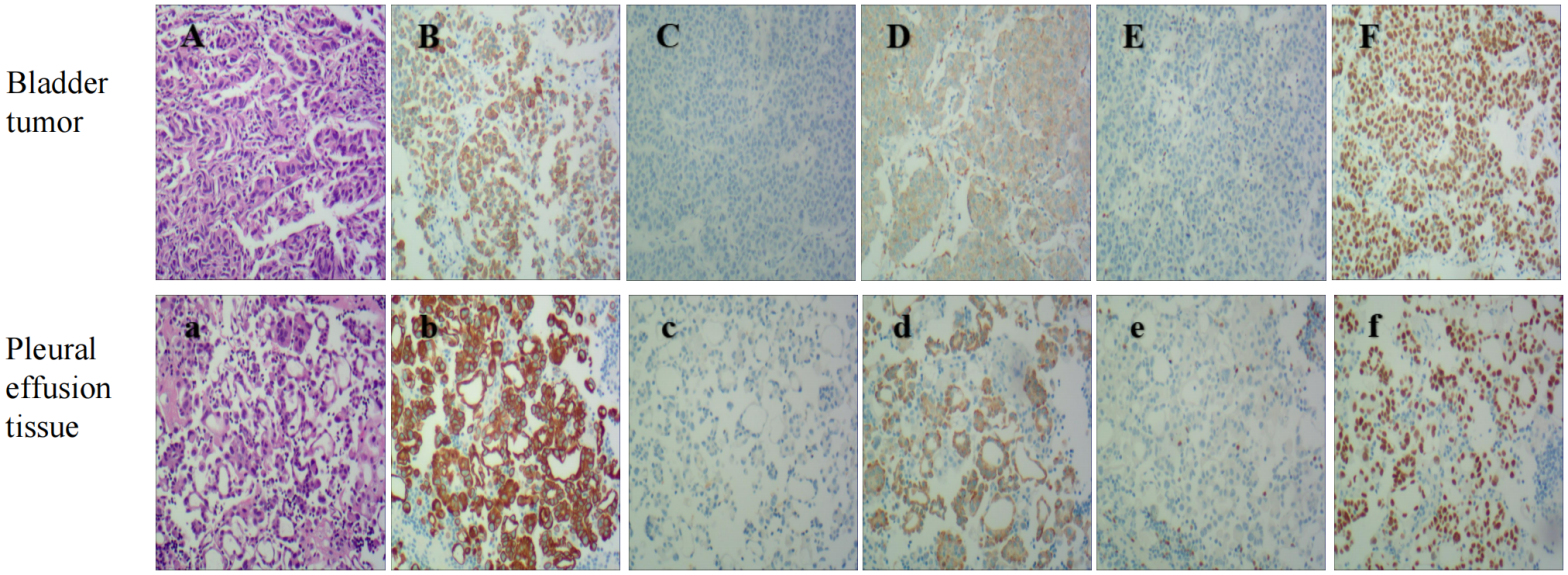
Bladder Tumor IHC: HE stain **(A)**, The panels mean that CK7 **(B)**, Napsin A **(D)**, and TTF-1 **(F)** are positive, While GATA3 **(C)**, pax8 **(E)** are negative(original magnification 100X). Pleural effusion tissue IHC: HE stain **(a)**. The panels mean that CK7 **(b)**, Napsin A **(d)**, and TTF-1 **(f)** are positive, While GATA3 **(c)**, pax8 **(e)** are negative(original magnification 100X).

**Figure 3:**
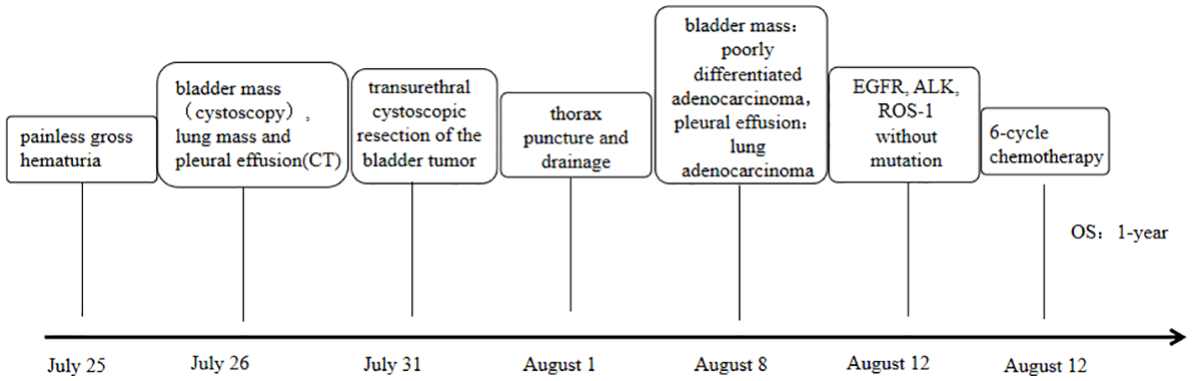
Timeline of clinical events.

The patient (female, 72 years old) was admitted to the outpatient clinic due to “gross hematuria for one day”. She had a history of hypertension and no family history of tumors. The patient developed hematuria without obvious inducement, which was characterized as full-course, painless, without obvious blood clots, with slightly frequent and urgent urination. After admission, relevant examinations were completed. Cystoscopy showed space-occupying lesions in the bladder. Chest CT examination suggested space-occupying lesions in the lower lobe of the lung, suggesting peripheral lung cancer with cervical, mediastinal and hilar lymph node metastases (**Figure 1**). There were multiple small nodules in both lungs with possible metastasis and a left pleural effusion. Possible diagnoses included primary lung cancer, primary bladder cancer, or bladder metastasis from lung cancer. To solve the hematuria problem, transurethral cystoscopic resection of the bladder tumor was performed. Pathological examination of the bladder mass indicated the following: poorly differentiated adenocarcinoma and a tendency toward lung adenocarcinoma metastasis based on combined clinical and immunohistochemistry findings. Immunohistochemistry revealed the following: CK7 (+), TTF-1 (+), NapsinA (+), CK20 (-), GATA3 (-), Syn (-), CgA (-), S-100 (-), PAX-8 (-), and Ki-67(+) of approximately 40% (**Figure 2**). For the left pleural effusion, puncture and drainage were performed and sent for pathology. Pathological examination of the pleural effusion cell block revealed tumor cells that were consistent with adenocarcinoma, and immunohistochemical lung markers TTF-1, NapsinA, and CK7 were all positive. The lung adenocarcinoma was possibly large. In view of the partial positivity of the immunohistochemical marker Villin and the crossover phenomenon, the gastrointestinal tract was further examined. The immunohistochemistry findings were as follows: tumor cells: CK7 (+); TTF-1 (+); NapsinA (+); Villin: partial cells (+); CK20: few cells (+); GATA3 (-); CR (-); CK5/6 (-); CD163 (-); and PAX-8 (-)(**Figure 2**). Given the patient’s medical history, pathological findings and immunohistochemistry, bladder metastasis of lung adenocarcinoma was considered. Color ultrasound-guided thoracentesis and drainage of pleural effusion were performed, and the bladder mass was submitted for genetic testing. The results showed that there were no genetic mutations in EGFR, ALK or ROS-1. According to the results of genetic testing, there was no corresponding targeted drug, so we administered 6 cycles of pemetrexed combined with carboplatin chemotherapy. The patient agreed to the treatment and achieved one-year survival (**Figure 3**), which was better than expected based on other studies.

## 3 Discussion

Among bladder malignancies, urothelial carcinoma is the most common histological type, whereas adenocarcinoma, squamous cell carcinoma, and small cell carcinoma are not as common and account for smaller proportions. Primary adenocarcinoma has been reported to account for 0.5-2% of epithelial malignancies of the bladder (1). Secondary bladder cancer accounts for approximately 2% of bladder cancers (2). BATES et al. retrospectively analyzed 282 cases of bladder metastasis, which accounted for only 2.3% of all bladder cancers. The most common sites of the primary lesion included the colon (21%), prostate (19%), rectum (12%) and cervix (11%), which were caused by direct invasion of adjacent organs. The most common primary lesions in cases of distant metastasis included the stomach (4.3%), skin (3.9%) and mammary gland (2.5%). Secondary bladder tumors were found in 2.8% of lung malignancies (squamous cell carcinoma, carcinoma, adenocarcinoma and small cell carcinoma), of which 4 cases (1.6%) were squamous cell carcinoma and only 1 case (0.4%) involved lung adenocarcinoma as the source. Metastasis of lung adenocarcinoma to the bladder is particularly rare (3). In another large autopsy study, 0.16% of lung cancers had bladder metastases. Among the cases reported in the literature in the past 15 years, 15 cases of lung cancer with bladder metastasis were reported (**Table 1**). Of these, 14/15 (93.3%) (with histological examination) were adenocarcinomas and 1 was a neuroendocrine carcinoma (4). This trend coincides with an increase in the incidence of adenocarcinoma over the past two decades, as genetic testing and targeted drugs have greatly extended survival, leading to a greater prevalence of metastasis (19).

**Table 1:** List of reported cases of lung metastases to the bladder.

Age gender	clinical manifestation	Past history	Cystoscopy examination	pathology	IHC/Genetic Testing
65 Male (4)	Hematuria, Urinary retention,	Lung cancer a year ago, radiotherapy, chemotherapy	bladder neck and trigone	Small cell neuroendocrine carcinoma	CK-7(+),SynA(+), TTF-1(+)
52 Male (5)	microscopic hematuria, dysuria	Right upper lobectomy 6 months ago	a mass at the top of the bladder	adenocarcinoma	CK-7(+),TTF-1(+), CK-20 (-),
66 Male (6)	Severe nephrosis in the kidney	Lung cancer stage IV , chemotherapy	left trigone bladder	poorly differentiated carcinoma	TTF-1(+),CK7(+), P63(+),CK20 (-),AR (-)
61 Male (7)	Hydronephrosis of left kidney	Lung cancer stage IIIa , chemotherapy	Thickening of the bladder wall	adenocarcinoma	EGFR19(+) T790M(-)
63 Female (8)	arthralgia	Lobectomy dissection performed 10 months ago	A 3cm solid lesion was located on the left side of the bladder wall	adenocarcinoma	CK7(+),S100P(+),CK20(-),GATA3(+),CDX-2(-), TTF-1(-),napsinA(-)
81 Female (9)	Abdominal pain, Abnormal renal function	Chemotherapy six months ago	normal bladder mucosa, external compression of the bladder cavity	Adenocarcinoma involves muscularis propria	TTF-1(-), CK7(+), NapsinA(+)
83 Male (10)	gross hematuria	initial symptom	diverticulum on right posterior wall of bladder	adenocarcinoma	CK20(-),CK7(+), TTF-1 (+),PSA(-),AR(-)
55 Male (11)	Frequent, urgent urination	Simultaneous discovery	the trigone and right lateral wall of the bladder	adenocarcinoma	CK7(+),TTF1(+), CK20(−),PSA(-)
73 Female (12)	gross hematuria	Lung cancer thirteen months ago	a mass in the trigone of the bladder		CK20(-),TTF-1 (+),P63(-), GATA(-),CK7(+),napsinA(+) **EGFR19(+),T790M(-)**
78 Male (13)	gross hematuria	Lung cancer stage IA Chemotherapy erlotinib	3mm papillary tumor in the right lateral wall of the bladder	adenocarcinoma Intact urothelium, myometrial invasion	TTF-1 (+), CK-7(+), CK-20(-)
65 Female (14)	gross hematuria	Lung cancer stage IV, Chemotherapy	solid lesions protrude into the bladder lumen	adenocarcinoma	TTF-1(+),CK7(+), CK20(-),CD15(-)
53 Male (15)	hematuria	Lung cancer with liver and brain metastases a year ago	the epithelium is intact, and the underlying detrusor muscle is infiltrated	adenocarcinoma	TTF-1 (+), CK-7(+), CK-20(-)
71 Male (16)	hematuria	Left inferior lobe mass two years ago	along the urinary tract without bladder muscle invasion	adenocarcinoma	CK-7(+),TTF-1(+), napsinA(+),CK20(-), PSA(-),P504S(-)
86 Female (17)	Frequent urination, urge incontinence	Left lung T2aN0M0 2 years ago, Radiation therapy	Nodular mass with calcification in the left parietal wall	adenocarcinoma The tumor has invaded the muscular layer of the bladder	TTF-1(+),CK7(+),CK20(-),napsinA(-),p53(+),GATA3(+), Uroplakin III(-)
40 Male (18)	hematuria	Left lung cancer with right femur metastasis 1 year ago		adenocarcinoma	TTF-1(+), CK7(+), CK20(+)

Primary bladder cancer usually presents with painless gross hematuria. Similarly, of the 15 cases reported, the most common clinical manifestation of bladder metastasis of lung cancer was gross hematuria (8/15 cases, 53.3%). Microscopic hematuria has also been reported, but it has only been reported in one case (5). Dysuria and urgency are other manifestations of bladder metastasis. In addition, 4 of 15 patients (26.7%) presented with hydronephrosis and bladder irritation symptoms such as urinary obstruction, pelvic pain, dysuria, oliguria, and anuria (4–7). Two patients (13%) with partial bladder cancer metastasis presented with arthralgia and abdominal pain (8, 9).

In the auxiliary examination, color ultrasound and CT often indicate focal or diffuse thickening of the bladder wall, which is difficult to distinguish from primary bladder cancer (10, 11). Li et al. reported a pedicled mass in the bladder wall, which was consistent with primary bladder cancer and difficult to distinguish (12). Therefore, if bladder metastasis is suspected, cystoscopy and transurethral resection (TUR) biopsy are necessary. Because primary bladder tumors are often multiple, some authors have suggested that cystoscopic visualization of a single lesion may be a clue to bladder metastasis. Francesca S et al. reported that 60% of cases of bladder metastasis from lung cancer involved a single lesion, 20% involved multiple lesions, and 20% involved intact bladder mucosa under cystoscopy (13). Secondary lesions are more common in the lateral wall of the bladder (70%) and less common in the dome (20%) and triangle (10%). As previously mentioned in this patient, there was a single cystoscopically identified lesion, which was different from those of primary bladder cancer (14).

The usefulness of urine cytology in the assessment of primary bladder cancer or bladder metastasis of lung cancer is controversial. Urine cytology can result in false negatives or false positives. Due to metastatic bladder cancer, tumor cells are deeply buried in the mucosa and often cannot emerge through urothelial infiltration and ulceration. Even if they break through the urothelium, the number of detectable cells is too small to indicate positive urine cytology, resulting in false negatives. Other reports have shown positive urine cytology in patients with lung cancer without significant urinary metastases. False positives may be associated with advanced disease and high tumor burden. The authors have tried to explain this phenomenon by hypothesizing that lung cancer cells were implanted in the renal cortex. However, cystoscopy was not available for all positive patients, so the possibility of microbladder metastases cannot be ruled out (15).

The gold standard for the diagnosis of metastatic bladder cancer is pathology. Bladder wall lesions can be found by cystoscopy, and pathological diagnosis can be made by biopsy. The differentiation is mainly between primary and metastatic bladder tumors. Microscopically, the presence of adenoid metaplasia or mucinous metaplasia of Brunns cell nests or adjacent epithelium in adenocarcinoma specimens after TUR suggests the possibility of a primary tumor, but caution is warranted. In the few cases previously reported, the finding of an intact epithelium on a bladder tumor was considered suggestive of a secondary lesion. Immunohistochemistry can further support a differential diagnosis (13).

CK7 and CK20 are the most widely used markers for predicting the primary site of metastatic adenocarcinoma. CK7 is a basic keratin that mainly exists in adult lung, breast, ovary and other tissues. Studies have shown that CK7 is also increased in ovarian adenocarcinoma and lung adenocarcinoma. Its sensitivity as a marker is higher. CK20 is an important biological marker of intermediate filaments in epithelial cells. It is positive in normal gastrointestinal epithelium and gastric fovea epithelium. CK20 is highly expressed in gastric cancer, cholangiocarcinoma, gallbladder cancer and pancreatic cancer. Low expression is observed in breast cancer, lung cancer and ovarian cancer. Thus, the combination of CK7 and CK20-related results may provide a preliminary diagnosis of the primary site. For example, CK7+/CK20+ or CK20- phenotypes suggest lung or bladder as sites of the primary lesion, and CK7-/CK20+ phenotypes suggest a gastrointestinal origin (although some colorectal cancers may express CK7 and some bladder tumors may be CK20+) (12). Thyroid transcription factor 1 (TTF-1), which is expressed in primary lung adenocarcinoma, is highly sensitive and specific. Including the analysis of TTF-1 can help to determine whether adenocarcinoma originates from the lung. TTF-1 is a sensitive marker for primary lung adenocarcinoma, but its specificity is significantly lower than that of Napsin A. There are no reports of primary bladder adenocarcinoma expressing TTF-1 (16). However, of the 15 reported cases of bladder metastasis from lung adenocarcinoma, TTF-1 was found to be negative in 2 of the bladder metastases but positive in 1 of the primary tumors. Napsin A is an aspartate protease that is substantially expressed in the lung and kidney. Some studies have suggested that Napsin A is a marker that is more sensitive and specific to primary lung adenocarcinoma than TTF-1, although its expression seems to be negatively correlated with tumor grade. Napsin A has been found to be positive in up to 89% of primary lung adenocarcinomas and is a marker of high diagnostic value. S100P, a member of the S100 protein family, was originally found to be expressed in the placenta and in 78% of bladder urothelial carcinomas. GATA3 is also a marker of urothelial differentiation, with a sensitivity of up to 67-90% and low specificity because it is widely expressed in ductal and lobar breast cancer. Therefore, to exclude urothelial origin, IHC is recommended (17). Other IHC markers, such as Syn and CgA, are neuroendocrine markers, and PAX8 is used to label metastatic lesions of female reproductive tract origin. In this case, CK7 (+), TTF-1 (+), NapsinA (+), CK20 (–) and GATA3 (-) suggested a high possibility of lung adenocarcinoma origin.

At present, there are few studies on the correlation between driver gene mutations and organ metastasis, and no unified conclusion has been reached (19). Among the 15 patients with bladder metastasis of lung cancer reported thus far, including the one provided by us, only 3 patients had genetic test results, including 2 patients with EGFR19 mutations and 1 patient with a negative genetic test. This small number of patients did not permit comparative analyses of possible mutational associations (20). Additionally, in our case, genetic testing was performed on the bladder tissue, and we could also perform genetic testing on lung tissue to improve accuracy and detect heterogeneity between primary and metastatic sites of lung cancer. Whether the transfer of organs is related to driving gene mutations remains one of the hot issues that urgently need to be addressed in the future.

Little information on the treatment of this unusual disease can be obtained from the 15 reported cases available. TUR was the only local treatment for 3 patients. One of these patients died after 6 months, another was alive after 3 months, and for the third patient, detailed follow-up data were not available. Three patients received carboplatin/gemcitabine-based chemotherapy. One was alive at five months, while the other two died at eight and nine months. One patient underwent radical cystectomy. Follow-up data were not available for the remaining cases. Of note, no patients received external radiation therapy, although this may be an option for patients with non-small cell lung cancer.

## 4 Conclusions

The organs most commonly involved in advanced lung cancer metastasis cases include the brain, lung, and liver. Regarding the involvement of independent organs, bladder metastasis of lung cancer is extremely rare, as is the clinical incidence of metastatic bladder tumors, so there is no unified clinical treatment standard, especially given that there are few pathological types of metastatic adenocarcinoma. The prognosis of lung cancer patients with bladder metastasis is poor. The differential diagnosis of secondary bladder cancer and non-transitional cell primary bladder cancer is of great clinical importance. Apart from the patient’s medical history, the most important factor is accurate pathological evaluation. Immunohistochemical examination is beneficial for distinguishing primary and metastatic bladder adenocarcinoma. Treatment of metastatic cancer of the bladder often requires a joint effort across multiple disciplines, such as general surgery, anorectal, gynecology, pathology and urology. Radical surgery or necessary palliative surgery can be performed according to the condition. We hope that this case analysis can improve the clinical understanding of metastatic bladder cancer and prevent missed diagnosis and misdiagnosis in clinical practice.

## Data availability statement

The original contributions presented in the study are included in the article/supplementary material. Further inquiries can be directed to the corresponding author.

## Ethics statement

Written informed consent was obtained from the individual(s) for the publication of any potentially identifiable images or data included in this article.

## Author contributions

XD, YL, and YX worked on the case and wrote the manuscript. XD, NL and TL participated in the collection of case data and literature, as well as the completion of all documentary and article work. YL and TL has given many constructive suggestions for this paper. All authors contributed to the article and approved the submitted version.
